# Outcome of Full-Thickness Macular Hole Surgery in Choroideremia

**DOI:** 10.3390/genes8070187

**Published:** 2017-07-21

**Authors:** Mays Talib, Leonoor S. Koetsier, Robert E. MacLaren, Camiel J.F. Boon

**Affiliations:** 1 Department of Ophthalmology, Leiden University Medical Center, 2333 ZA Leiden, The Netherlands; m.talib@lumc.nl (M.T.); l.s.koetsier@lumc.nl (L.S.K.); 2 Nuffield Laboratory of Ophthalmology, University of Oxford and Oxford Eye Hospital, Oxford University Hospitals NHS Foundation Trust, Oxford OX3 9DU, UK; enquiries@eye.ox.ac.uk; 3 Department of Ophthalmology, Academic Medical Center, 1000 AE Amsterdam, The Netherlands

**Keywords:** *CHM*, choroideremia, macular hole, vitrectomy

## Abstract

The development of a macular hole is relatively common in retinal dystrophies eligible for gene therapy such as choroideremia. However, the subretinal delivery of gene therapy requires an uninterrupted retina to allow dispersion of the viral vector. A macular hole may thus hinder effective gene therapy. Little is known about the outcome of macular hole surgery and its possible beneficial and/or adverse effects on retinal function in patients with choroideremia. We describe a case of a unilateral full-thickness macular hole (FTMH) in a 45year-old choroideremia patient (c.1349_1349+2dup mutation in *CHM* gene) and its management. Pars plana vitrectomy with internal limiting membrane (ILM) peeling and 20% SF_6_ gas tamponade was performed, and subsequent FTMH closure was confirmed at 4 weeks, 3 months and 5 months postoperatively. No postoperative adverse events occurred, and fixation stability improved on microperimetry from respectively 11% and 44% of fixation points located within a 1° and 2° radius, preoperatively, to 94% and 100% postoperatively. This case underlines that pars plana vitrectomy with ILM peeling and gas tamponade can successfully close a FTMH in choroideremia patients, with subsequent structural and functional improvement. Macular hole closure may be important for patients to be eligible for future submacular gene therapy.

## 1. Introduction

Choroideremia (CHM) is an X-linked recessive, progressive degeneration of the retinal pigment epithelium (RPE), outer retina, and choroid. It is caused by mutations in the *CHM* gene, which encodes the Rab escort protein 1 (REP1). Affected males characteristically experience night blindness in the first decade of life, a progressive visual field restriction in early adulthood, and slow visual acuity (VA) loss until the fifth and sixth decade of life, after which VA may decrease more rapidly [1].

Few studies have reported cases of a macular hole in CHM patients, which may occur in up to 10% of patients with advanced CHM [2,3]. Little is known about the outcome of macular hole surgery and possible beneficial and/or adverse effects of vitrectomy surgery and inner limiting membrane (ILM) peeling on retinal function. Here, we describe the clinical findings and outcome of pars plana vitrectomy with ILM peeling and gas tamponade in a CHM patient with a full-thickness macular hole (FTMH).

## 2. Materials and Methods

We describe a patient with a clinical diagnosis of CHM established at the Department of Ophthalmology of Leiden University Medical Center (LUMC), a tertiary referral center for inherited retinal diseases. Sanger sequencing of genomic DNA revealed a hemizygous pathogenic mutation in the *CHM* gene (NM_000390.2; c.1349_1349+2dup), genetically confirming CHM. The last full-field electroretinogram was performed at the age of 19, showing a rod-cone pattern, with remaining rod responses of 10–15% of the normal values, and remaining cone responses of 40–50% of the normal values.

The patient provided written informed consent for the publication of the genetic and clinical data. The informed consent was signed within the framework of the RD5000 consortium, a Dutch national consortium for the registry of patients with inherited retinal dystrophies [4], as approved by the Medical Ethics Committee of the Erasmus Medical Center (MEC-2010-359), and as locally approved by the Institutional Review Board at the LUMC (ID P11.100). Patient examinations were performed following best-practice guidelines and equipment at the LUMC.

To close the full-thickness macular hole, 23-gauge pars plana vitrectomy was performed under general anaesthesia, by one of the authors (C.J.F.B.), with creation of a triamcinolone-assisted posterior vitreous detachment, subsequent epiretinal membrane (ERM) and ILM peeling using a 23-gauge end-gripping forceps (Bausch & Lomb Incorporated, Rochester, NY, USA) and a plano-concave silicone magnifying lens (FCI Ophthalmics, Paris, France), after staining the ERM and ILM with Membrane Blue Dual^TM^ dye (D.O.R.C. International, Zuidland, The Netherlands), consisting of 0.15% trypan blue, 0.025% brilliant blue G, and 4% PEG (polyethylene glycol), to stain the ERM and ILM. Postoperative face-down positioning was prescribed for 5 days.

## 3. Results

### 3.1. Preoperative Presentation

**Figure 1:**
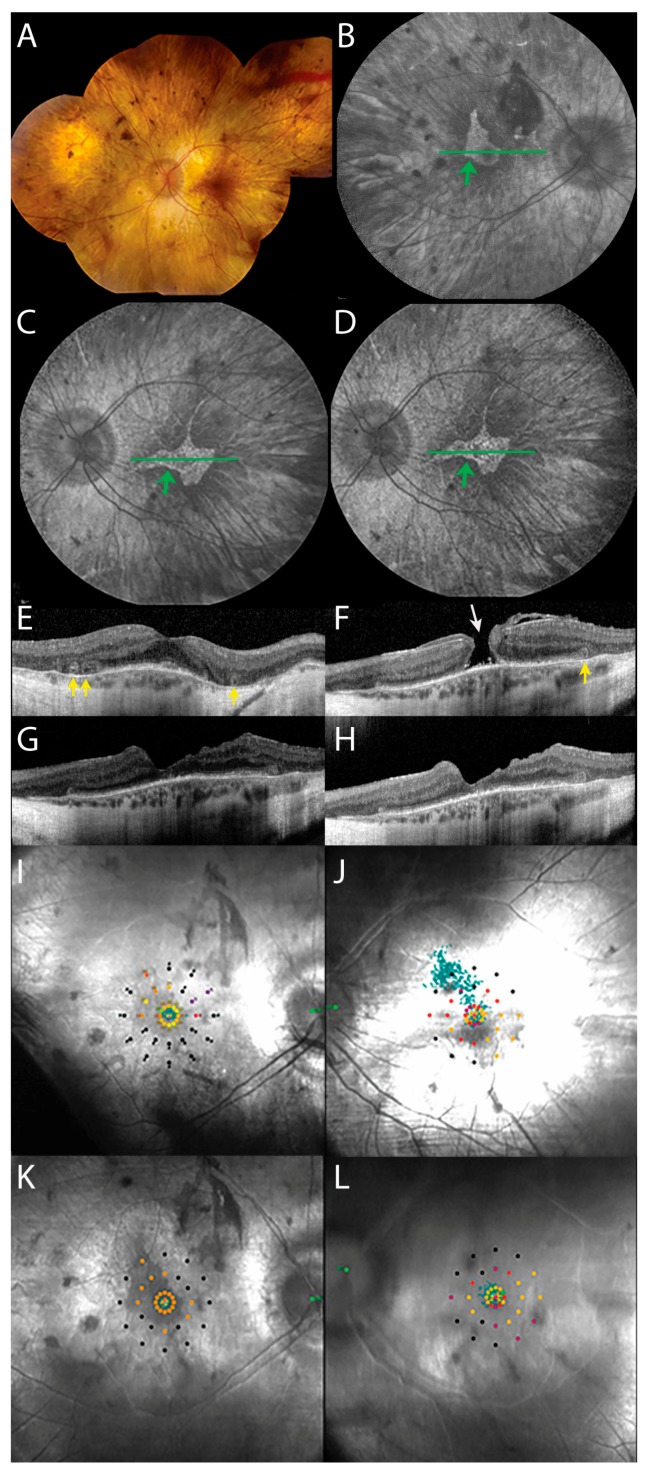
Imaging and functional findings before and after macular hole surgery in a choroideremia patient with a full-thickness macular hole. (**A**) Preoperative fundus photography of the left eye, showing bilateral atrophy of the retinal pigment epithelium (RPE), vascular attenuation, and waxy pallor of the optic discs. (**B** and **C**) Pre-operative fundus autofluorescence image of the right (**B**) and left (**C**) eye, showing relatively preserved RPE, with a sharply demarcated central island of relative preservation (green arrows), encompassing a temporal juxtafoveal hypo-autofluorescent lesion. (**D**) Postoperative fundus autofluorescence image of the left eye, showing no changes other than a mild deepening of the juxtafoveal hypo-autofluorescent lesion. The green horizontal lines in the fundus autofluorescence images (**B**–**D**) show the location of the complementary spectral-domain optical coherence tomography (SD-OCT) scans (**E**–**H**). (**E**) SD-OCT scan of the right eye, showing relative foveal preservation of the retinal layers in an atrophic retina, and outer retinal tubulations (yellow arrows). (**F**) Pre-operative SD-OCT of the left eye, showing a full-thickness macular hole (white arrow). Other central retinal abnormalities include an outer retinal tubulation (yellow arrow). (**G** and **H**) SD-OCT scans of the left eye, confirming closure of the macular hole 3 (**G**) and 5 (**H**) months postoperatively, and showing an altered temporal foveal contour, which is a common post-operative occurrence. (**I** and **J**) Preoperative microperimetry of the right (**I**) and left (**J**) eye, showing eccentric fixation in the left eye as compared to the right eye, as indicated by the cloud of green fixation points. (**K** and **L**) Microperimetry of the right (**K**) and left (**L**) eye, 5 months postoperatively, showing markedly improved fixation stability and sensitivity in the left eye, as the cloud of green fixation points spans a smaller and more centrally located area. The scotoma inferior to the final fixation locus mildly deepened post-operatively.

A 45-year-old man with a history of CHM, clinically diagnosed at 11 years of age, was referred to our hospital by the general practitioner for referral to a visual rehabilitation centre. He had complaints of nyctalopia and visual field restriction, mainly in the periphery, but increasingly in the central visual field. The visual acuity of his left eye had always been worse than his right eye due to amblyopia, and had been fluctuating around 20/50–20/67 in the last decade. He had noticed a decline in subjective visual acuity for at least a few months, with no clear predominance in one eye. The best-corrected visual acuity (BCVA) was 20/20 in the right eye, and 20/100 in the left eye, with a spherical refractive error of −1.75 D and −2.75 D in the right and left eye, respectively, and an astigmatism of −0.75 D in both eyes. Fundoscopy showed extensive chorioretinal atrophy in the posterior pole and the retinal periphery in both eyes, with irregularly-shaped islands of residual relatively preserved retina in the macula, with a full-thickness macular hole in the left eye (Figure 1A). Goldmann visual fields showed bilateral nasal peripheral visual field constriction with a midperipheral scotoma (Supplementary Materials Figure S1).

Fundus autofluorescence images (Spectralis, HRA, Heidelberg Engineering) showed a marked decrease of autofluorescence, with a central area of relative RPE preservation. Inside this island of relative RPE preservation, a juxtafoveal hypo-autofluorescent lesion, indicative of RPE change, could be seen in both eyes (Figure 1B–D).

A spectral-domain optical coherence tomography (SD-OCT) scan (Heidelberg Engineering, Heidelberg, Germany) showed typical CHM features in both eyes (Figure 1E,F), including extrafoveal atrophy of the external limiting membrane, ellipsoid zone, RPE, and choroid, and confirmed the full-thickness macular hole in the left eye, with a mild epiretinal membrane, and mild secondary parafoveal retinal thickening (Figure 1F). The right eye showed relative sparing of the macular structures without evidence of (impending) macular hole. Outer retinal tubulations were visible on the transitional zone between atrophic and relatively preserved areas of ellipsoid zone in both eyes. This distance between the fovea and the transitional zone could not be reliably determined in the left eye, but was 770 µm temporally and 605 µm nasally in the right eye.

Preoperative microperimetry (MAIA, CenterVue, Padova, Italy) showed reduced central retinal sensitivity in both eyes (supra-threshold testing in the left eye: 0% ≥ 25 dB; 32% 15–25 dB range) and eccentric fixation in the left eye (unstable fixation; 11% and 44% of fixation points located within a distance of 1° and 2°, respectively) as compared to the right eye (stable fixation; 100% of fixation points located within a distance of 1°).

The patient underwent 23-gauge pars plana vitrectomy under general anaesthesia, followed by postoperative face-down positioning for 5 days. No preoperative or postoperative complications occurred.

### 3.2. Postoperative Clinical Course

According to the patient, the gas bubble had disappeared after 3.5 weeks. After gas reabsorption, SD-OCT confirmed full-thickness macular hole closure at 1, 3, and 5 months postoperatively, with some inner retinal dimpling and an altered temporal foveal contour at 3 and 5 months (Figure 1G,H). No objective visual acuity improvement was achieved, with a BCVA of 20/133 to 20/100 in the left eye, but both the patient and his partner reported a mild subjective visual acuity improvement and less distorted near vision 3 months after surgery. Autofluorescence images (Spectralis HRA, Heidelberg Engineering) did not show postoperative changes as compared to the preoperative situation (Figure 1C,D), other than a mild deepening of the juxtafoveal hypo-autofluorescent lesion within the central island of relative RPE preservation. The size of this central area of relative preservation of autofluorescence, as delineated with the Heidelberg Spectralis Region Finder tool, did not decline postoperatively (preoperative 4.53 mm^2^; 5 months postoperative 4.8 mm^2^).

Compared to preoperative microperimetry (Figure 1I,J), microperimetry 5 months postoperatively showed an improvement in the macular sensitivity (supra-threshold testing in the left eye: 3% ≥ 25 dB; 41% 15–25 dB range), and an improvement in fixation stability in the operated eye (stable fixation; 94% and 100% of fixation points located within a distance of 1° and 2°, respectively; Figure 1K,L). Postoperative Goldmann visual fields did not show changes in the operated eye (Supplementary Materials Figure S1), and the seeing retinal area for all investigated isopters, as measured with the Field Digitizer [5], did not decline (preoperative 354.97 mm^2^ for the V4e; 5 months postoperative 369.9 mm^2^ for the V4e).

## 4. Discussion

Gene therapy for CHM patients has been described to be safe and potentially effective, using an adeno-associated viral vector to deliver *CHM* via a submacular injection using a pars plana vitrectomy approach [6]. However, as many as 10% of advanced CHM patients may develop a macular hole, possibly due to increased vulnerability of the fovea to anteroposterior vitreous traction, tangential tractional forces of the ILM/epiretinal membrane, and/or chronic low-grade inflammation resulting from outer retinal cell death. A macular hole will preclude successful submacular viral vector delivery in the context of gene therapy in these patients [2].

We described a FTMH in a patient with genetically confirmed CHM, which was successfully managed with pars plana vitrectomy, and ERM and ILM peeling, leading to a mild subjective improvement as well as improvement of retinal structure and function on OCT and microperimetry, respectively. The postoperative abnormal temporal foveal contour is a common occurrence following macular hole surgery [7], and may point to mechanical trauma, or to changes in the retinal microstructural stability due to ILM peeling. Central structural abnormalities on SD-OCT associated with CHM in this patient included outer retinal tubulations and a transition zone in relatively close proximity to the foveal centre, both of which have generally been noted to dictate caution when considering potential gene therapy trial inclusion [8], as mechanical trauma may further damage the already fragile photoreceptors. Gas resorption in CHM patients may be slower than usual due to extensive atrophy of the choroid. The duration until complete 20% SF_6_ gas tamponade resolution of 4 weeks in our case was longer than the previously reported median of 2 weeks in a macular hole patient population without CHM [9], but shorter than the 6 weeks described in a previously described CHM patient with macular hole, who received gas tamponade with 30% SF_6_ [2]. In this earlier case series of 3 CHM patients, gas tamponade with either 16% C_3_F_8_ or 30% SF_6_ generally lasted approximately twice as long as expected in standard FTMH surgery, which is consistent with our findings. We found no postoperative visual acuity decline or any adverse effects on autofluorescence, microperimetry, and Goldmann peripheral visual field testing during a 5-month follow-up period. One case report described a successful vitrectomy in a CHM patient with retinal detachment due to a macular hole, with postoperative macular hole closure, retinal reattachment, and improvement of subjective symptoms [3]. In the current case report, we show that an FTMH in CHM can be successfully closed with improvement in retinal function and without adverse effects. Such surgical FTMH closure may enable future treatment with gene therapy. The incidence of macular hole reopening after initial closure has been shown to be less than ten percent [10], and repeatedly less than one percent [11,12], in large study populations of patients without known inherited retinal dystrophies. However, it is currently unclear if CHM patients with a history of an FTMH are still more vulnerable to macular damage and reopening of the macular hole after submacular injection of gene therapy vector, despite the previous surgical closure of the macular hole.

## Supplementary Materials

The following are available online at www.mdpi.com/2073-4425/8/7/187/s1. Figure S1: Goldmann visual fields before and after macular hole surgery in a choroideremia patient with a full-thickness macular hole. (**A** and **B**) Preoperative Goldmann visual fields of the right (**A**) and left (**B**) eye, showing bilateral nasal peripheral visual field constriction with a midperipheral scotoma. (**C** and **D**) Postoperative Goldmann visual fields showing no significant changes.
