# Supplementary material for: Outcome of Full-Thickness Macular Hole Surgery in Choroideremia
Source: Genes (Basel). 2017 Jul 21;8(7):187. doi: 10.3390/genes8070187 (PMC5541320; doi:10.3390/genes8070187)
Supplement: Supplementary file 1 [file genes-08-00187-s001.pdf]

Supplementary Materials: Outcome of Full-Thickness Macular Hole Surgery in Choroideremia

Mays Talib, Leonoor S. Koetsier, Robert E. MacLaren and Camiel J.F. Boon

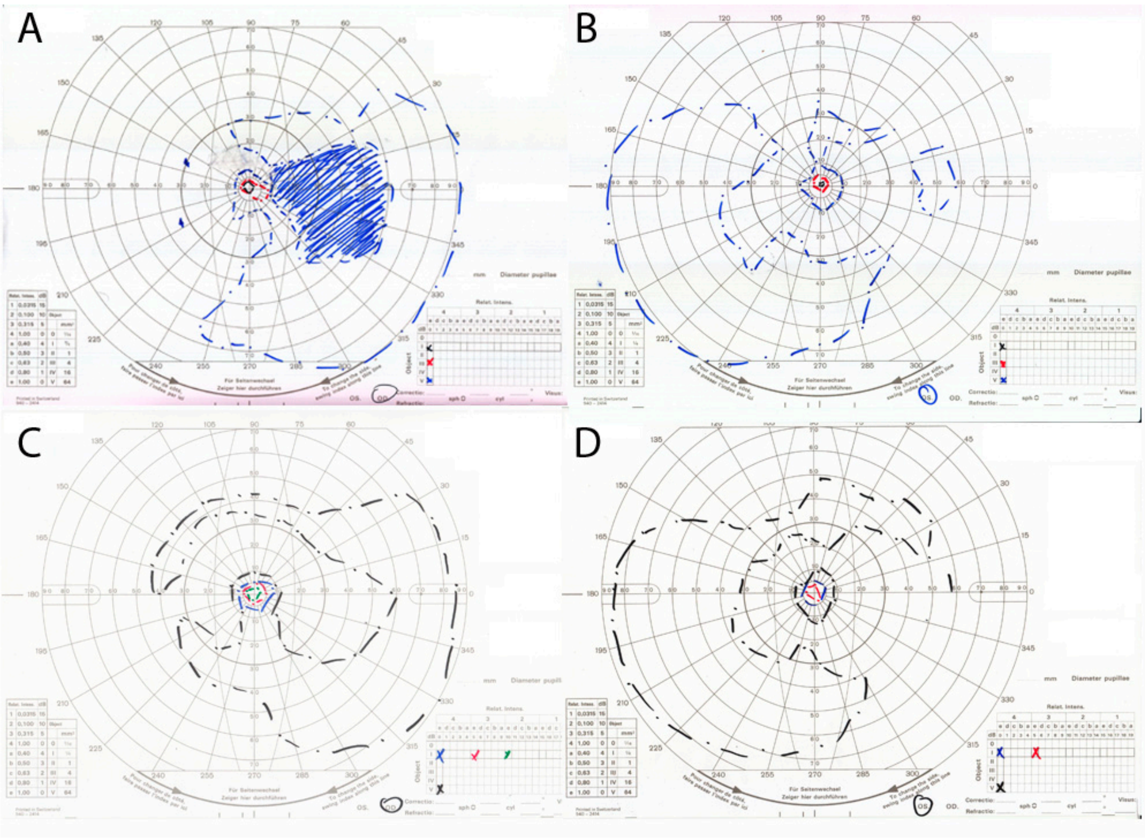

Figure S1. Goldmann visual fields before and after macular hole surgery in a choroideremia patient with a full-thickness macular hole. (**A** and **B**) Preoperative Goldmann visual fields of the right (**A**) and left (**B**) eye, showing bilateral nasal peripheral visual field constriction with a midperipheral scotoma. (**C** and **D**) Postoperative Goldmann visual fields showing no significant changes.
